# Functional Outcome after Simultaneous Bilateral Four-Part Proximal Humerus Fracture: A Comparison of ORIF and Hemiarthroplasty in an Individual Patient

**DOI:** 10.1155/2012/941829

**Published:** 2012-08-29

**Authors:** Prasad Ellanti, Paul Harrington

**Affiliations:** Department of Orthopaedics, Our Lady of Lourdes Hospital, Drogheda, Ireland

## Abstract

Simultaneous bilateral four-part proximal humeral fractures are rare. Four-part fractures of the proximal humerus are difficult for the patient and technically demanding for the surgeon. Our surgical tactic is to attempt open reduction and internal fixation where possible. We report the functional outcome in a 56-year-old female who sustained simultaneous bilateral four-part proximal humeral fractures after falling down a flight of stairs. Open reduction and internal fixation using threaded pins and tension band suture was performed on one side, and shoulder replacement hemiarthroplasty was required on the other. Functional assessment was undertaken at two years after surgery, using the Oxford Shoulder Score. Although the objective outcomes assessment revealed little difference, the patient herself expressed a preference for the side treated by internal fixation. We conclude that an attempt to retain the native humeral head and the surgical tactic that favours internal fixation where possible is appropriate in these injuries. Excellent function can be achieved following hemiarthroplasty for trauma in a fit patient.

## 1. Introduction

Four-part proximal humeral fractures are uncommon and constitute around 3% of all proximal humeral fractures [1]. They are complex and difficult to manage with treatments ranging from conservative, internal fixation, or hemiarthroplasty and often with a poor outcome. Simultaneous bilateral proximal humeral fractures are rare and are usually associated with a dislocation secondary to a seizure episode and to lesser extent electrocution. Bilateral four-part proximal humerus fractures are rarer still with only a few reports published previously with all of them caused by a seizure [2] or electrocution [3]. We present a 56-year-old female patient that sustained a bilateral simultaneous four-part humeral fracture secondary to falling down a flight of stairs. Internal fixation was undertaken on one side and hemiarthroplasty on the other.

## 2. Case Report

**Figure 1:**
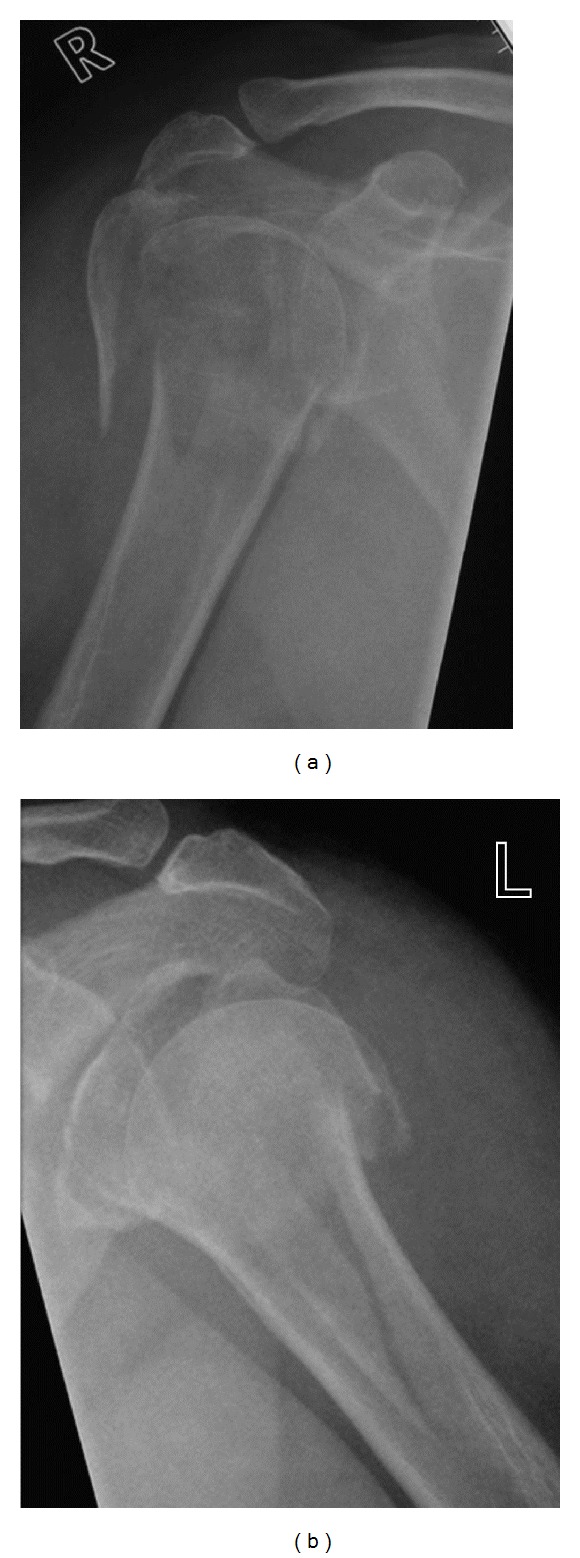
Preoperative radiographs demonstrating four-part proximal humeral fractures bilaterally.

A 56-year-old right-handed female office worker presented to the emergency department with marked pain and limitation of movement to both shoulders subsequent to a fall down a flight of stairs at home. Minor bruising was noted around both shoulders. There were no neurovascular deficits. No other injuries were obvious on clinical examination. Plain film radiographs demonstrated bilateral proximal humeral fractures (Figure 1). The patient had no significant past medical history of note.

**Figure 2:**
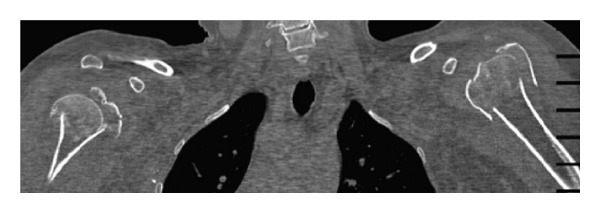
Coronal view of the CT of both shoulders demonstrating four-part proximal humeral fractures bilaterally.

**Figure 3:**
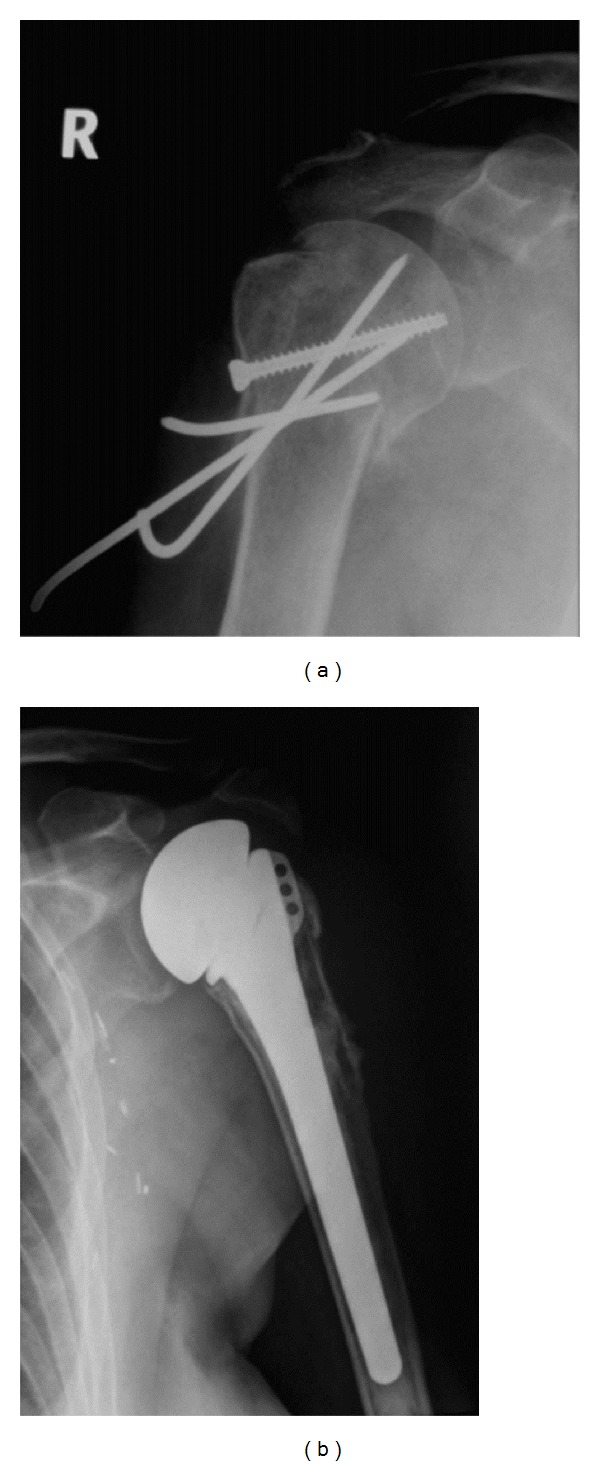
Postoperative radiographs demonstrating internal fixation with 2.7 mm threaded wires and figure of 8 tension band sutures using number 5 Ethibond suture on the right side and a hemiarthroplasty on the left side.

**Figure 4:**
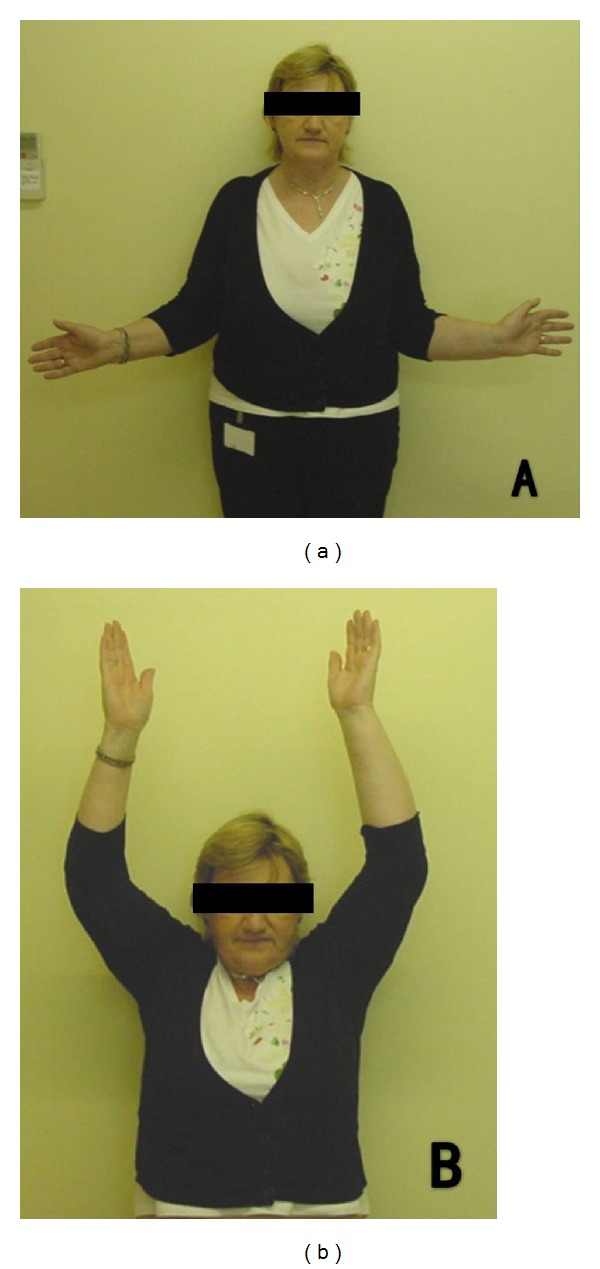
Photographs demonstrating active range of motion at two years postoperatively.

A CT scan confirmed bilateral four-part proximal humerus fractures (Figure 2). Open reduction and internal fixation with threaded pins and tension band suture was undertaken on the dominant right side. Seven days later, surgery on the left was performed. As a satisfactory open reduction could not be achieved, a replacement hemiarthroplasty was performed (Figure 3). The postoperative course was uneventful. At the two year followup, the patient reported an oxford shoulder score of 58/60 on the fixed right side and 57/60 on the replaced left side. The patient had an abduction of 150° bilaterally and near normal external rotation (Figure 4). Though there was little difference objectively, the patient expressed a preference for the shoulder which had been treated with internal fixation, stating that “it feels more natural and secure.”

## 3. Discussion

Bilateral four-part proximal humeral fractures are rare. They are complex injuries that are difficult to manage and often associated with a poor outcome. Several surgical techniques include percutaneous Kirshner wiring, tension-band wiring, transosseous suturing, screw fixation, plate fixation, intramedullary nail fixation, hemiarthroplasty, or, most recently, angle-stable plates. The optimal treatment of these fractures is controversial with only a few small randomised trials comparing treatments. Stableforth [4] showed results favouring arthroplasty when compared to nonoperative management in 32 patients with four-part proximal humeral fractures. Hoellen et al. [5] found no statistically significant difference in outcome in their study comparing arthroplasty with fixation in 30 patients. A systematic review by Misra et al. [6] showed that conservatively managed patients had a poor outcome; however, there was no significant difference between arthroplasty and fixation. They concluded that there was insufficient data available in literature to make evidence-based recommendations. Similar conclusion was reached in a subsequent Cochrane review of proximal humeral fractures [7].

It has been shown that most surgeons prefer fixation in the younger patient but there was little consensus with regard to the management of elderly patients. In our patient, the right dominant side was openly reduced and fixed. The left side was treated with a hemiarthroplasty after attempted open reduction. The functional outcome at two years was similar for both sides. This is in keeping with the available literature comparing fixation to arthroplasty. However, the patient preferred the right shoulder as it felt more natural.

## References

[1] Court-Brown CM, Garg A, McQueen MM (2001). The epidemiology of proximal humeral fractures. *Acta Orthopaedica Scandinavica*.

[2] Claro R, Sousa R, Massada M, Ramos J, Lourenço JM (2009). Bilateral posterior fracture-dislocation of the shoulder: report of two cases. *International Journal of Shoulder Surgery*.

[3] Cooke SJ, Hackney RG (2005). Bilateral posterior four-part fracture-dislocations of the shoulders following electric shock: a case report and literature review. *Injury Extra*.

[4] Stableforth PG (1984). Four-part fractures of the neck of the humerus. *Journal of Bone and Joint Surgery*.

[5] Hoellen IP, Bauer G, Holbein O (1997). [Prosthetic humeral head replacement in dislocated humerus multi-fragment fracture in the elderly—an alternative to minimal osteosynthesis?]. *Zentralblatt Fur Chirurgie*.

[6] Misra A, Kapur R, Maffulli N (2001). Complex proximal humeral fractures in adults—a systematic review of management. *Injury*.

[7] Handoll HH, Gibson JN, Madhok R (2002). Interventions for treating proximal humeral fractures in adults. *Cochrane Database of Systematic Reviews*.

